# The prevalence of pulp stones in a Turkish population. A radiographic survey

**DOI:** 10.4317/medoral.17400

**Published:** 2011-12-06

**Authors:** Yıldıray Sisman, Ali M. Aktan, Elif Tarım-Ertas, Mehmet E. Çiftçi, Ahmet E. Şekerci

**Affiliations:** 1 PHD, DDS. Department of Oral Diagnosis and Radiology, School of Dentistry, Erciyes University, Kayseri, Turkey PHD, DDS Deparment of Oral Diagnosis and Radiology, School of Dentistry, Katip Çelebi University, İzmir; 2 PHD, DDS. Department of Oral Diagnosis and Radiology, School of Dentistry, Gaziantep University, Gaziantep, Turkey; 3 PHD. Department of Oral Diagnosis and Radiology, School of Dentistry, Gaziantep University, Gaziantep, Turkey; 4 PHD. Department of Oral Diagnosis and Radiology, School of Dentistry, Erciyes University, Kayseri, Turkey

## Abstract

Objectives: The goal of this retrospective study was to determine the prevalence of pulp stones in a Turkish population. Any possible associations between pulp stones and gender, tooth type and dental arch were also evaluated. Study Design: Four hundred and sixty nine patients’ bitewing radiographs which were reached through the patient database of Erciyes University Dentistry School, Department of Oral Diagnosis and Radiology were examined. Of these 469 subjects whose mean age was 24( ± 10.7), 302 were females and 167 were males. A total of 6,926 teeth were examined during this study. Pulp stones were recorded as present or absent and any relations with gender, tooth type and dental arch were noted. Results: Pulp stones were identified in 270 (57.6 %) of the subjects and in 1,038 (15 %) of the teeth examined. Their presence were seldom found in the premolars (9.07%) but was much higher in the molars (90.92 %). Pulp stone occurrence was significantly more common in the first molars than in the second molars, and in the first premolars than in the second premolars in each dental arch. Their occurrence was higher in the maxilla than in the mandible for each tooth type. No difference between the two genders could be identified. Conclusion: Pulp stones are not only incidental radiographic findings of the pulp tissue but may also be an indicator of some serious underlying disease. On the other hand, they may provide useful information to predict about the susceptibility of patients for other dystrophic soft tissue calcifications such as urinary calculi and calcified atheromas. However, further study on this issue is needed.

** Key words:** Prevalence, pulp stone, Turkish population.

## Introduction

Pulp stones are calcified bodies in the dental pulps of the teeth in the primary and permanent dentition. They can be seen in the pulps of healthy, diseased, and even unerupted teeth (1). Their locations are more common in the coronal than in the radicular portions of the pulp and they can be observed as free, attached, and embedded in the dentinal surface of the pulp chamber. Pulp stones are classified done according to their structure as true, false, and diffuse. They range in size from small microscopic parti-cles to large masses that almost obliterate the pulp chamber (2). 

Although the exact cause of pulp calcification is unknown some factors have been implicated in stone formation such as genetic predisposition (3), orthodontic tooth movement, circulatory disturbance in pulp, age (4), interactions between the epithelium and pulp tissue, idiopathic factors (5), and long-standing irritants like caries, deep restorations, and chronic inflammation (6). Studies related to the prevalence of pulp stones, based on radiographic examinations, have been reported with various percentages (rang-ing from 8% to 95%) (7-16). Females are more frequently affected than males (11,16,17). However, to our knowledge, there has been only one report on the prevalence of pulp stone involving a Turkish population (16).

The aim of this radiographic-based study was to determine the prevalence of pulp stones in a group of Turkish population, and to evaluate possible associations between pulp stones and gender, tooth type, dental arch, and side, and to compare the results with published data presenting a new perspective in forensic medicine.

## Material and Methods

The comprised bitewing radiographic-based materials which were examined in the present study were collected from the 8000 files of patients who were referred to Department of Oral Diagnosis and Radiology at Erciyes University’s Faculty of Dentistry, for routine dental examination. Patients whose bitewing radiographs were taken bilaterally during routine radiographic examination were included in the present study. After examining patients’ data those with crown, bridge, and deep restoration were excluded from the study, of those patients with bilateral bitewing radiographs, a total of 469 patients, 302 female and 167 males, were included in the present study.

Only the maxillary and mandibular molars (wisdom teeth were excluded) and premolars were included. Subjects with crowns or bridges that prevented adequate vision of the pulp chamber were not included in the study sample. Considering that teeth with deep fillings and caries lesions are more inclined to have pulp stones, only teeth which were non-carious and unrestored, or those with shallow fillings, were included. The radiographs were interpreted by two examiners using a standard viewing box and under subdued ambient light. Those taken at the wrong angulation, in inappropriate exposure and processing faults were excluded.

**Figure 1:**
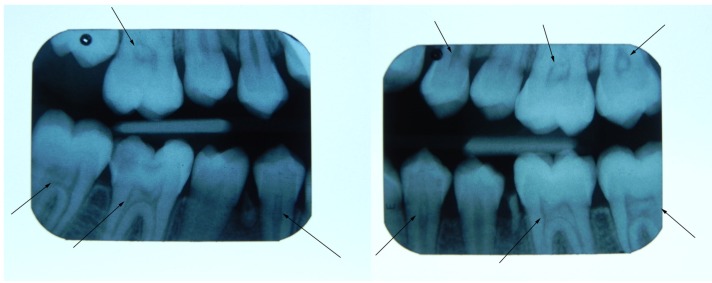
Pulp stone observed inside the pulp chambers of the molars and premolars in the bitewing radiograph.

Definite radiopaque bodies observed inside the pulp chambers of the molars and premolars were identified as pulp stones (Fig. 1) and were scored as present or absent. No attempt was made to determine the details of the pulp stones, such as their number, size and location in the pulp chamber. 

To ensure of the accuracy of the diagnosis, only the teeth that were confirmed by our two examiners to have pulp stones were scored as present. Those teeth about which both our examiners were unsure were re-examined by our senior dental radiologist and scored according to his diagnosis. Additionally, every day during the study our senior dental radiologist randomly selected 5 ra-diographs

from among those that were examined by our two examiners and compared his own diagnosis with theirs.

Descriptive statistics were determined, including the patient’s age and gender, and the location, type, and arch of the pulp stones. Differences between the gender and tooth type for the distribution of the pulp stone were compared by the Mann-Whitney U Test. The occurrence of the pulp stones according to location, side and arches were compared by Wilcoxon Signed Rank Test. The Cohen’s Kappa was calculated to examine inter-examiner repeatability for evaluation of the pulp stones. All Analyses were performed by SPSS statistics program for windows version (SPSS version 11.0, SPSS Inc., Chicago, IL, USA). 

## Results

A total of 469 patients (302 females and 167 males) participated in the present study. The age range of the subjects was 10 to 59 and the mean age was 24.74 (± 10.7).

Of the 469 subjects, pulp chamber calcifications were observed in 270 patients; 176 in women and 94 in men. Fifty four patients (11.5%) had only one tooth with a pulp chamber calcification, while in 216 patients (24%) more than one tooth was affected. In addition, in the bitewing radiograph of one male patient 16 teeth were detected with pulp chamber calcification.

In total 6,926 teeth were examined during the study. In this randomly selected study, 1,038 teeth with pulp stones in the pulp chamber or coronal portion of the roots were detected, 731 in those of females and 307 were in those of males.

The incidence of pulp stone was found to be 15 %. Pulp stones were detected in 731 of the 4,479 teeth (10.56 %) examined in females and in 307 of the 2447 teeth (4.44 %) examined in males with significant difference between the genders (p<0.05) ( Table 1). The occurrence of pulp stones was higher in the maxilla than in the mandible in each tooth type and when data for both arches were combined (p<0.001) ( Table 2). There were no statistically significant differences between the right and the left side in each tooth type and arch (p=0.101) ( Table 3). Pulp stones were found in only 96 (9.07 %) of the 3538 premolars and in 962 (90.92 %) of the 3424 molars examined, with differences in occurrence being statistically significant (p<0.001). The frequency of pulp stones was higher in the first molars than in the second molars and in first premolars than in second premolars in each dental arch and when data for both arches were combined (p<0.001) ( Table 4). 

## Discussion

Calcification in the dental pulp can lead to denticles, commonly known as pulp stones. Therefore, the term “pulp stone” was used to indicate pulpal calcification in the present study. They are often incidental findings on dental radiographs and in the literature the incidence of pulp stones has been investigated in many radiological studies (7,8,11,15,16,18). In such studies bitewing and periapical radiographs were used and it was stated that these two radiographic techniques did not show significant differences in the diagnosis of pulpal calcification (15,19,20). However, since the bitewing radiographic technique is a better radiographic method rather than other conventional techniques (7), it was used in this study to illustrate the pulpal anatomical structure accurately.

When the literature related to pulp stones was reviewed, there were a limited number of studies regarding the incidence of pulp stones. Moreover, the reported rates of prevalence also differed in the studies. Some researchers reported prevalence based on the number of patients and teeth, whereas the others represented only the rates based on teeth numbers (7,11,16). In the present study, we

**Table 1:**
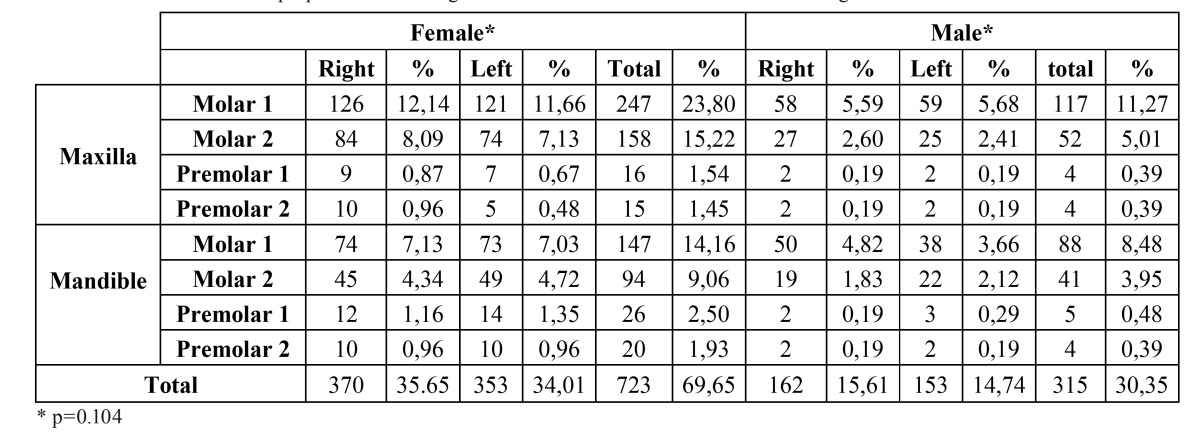
The distribution of pulp stone according to dental arches and their locations in each gender.

**Table 2:**
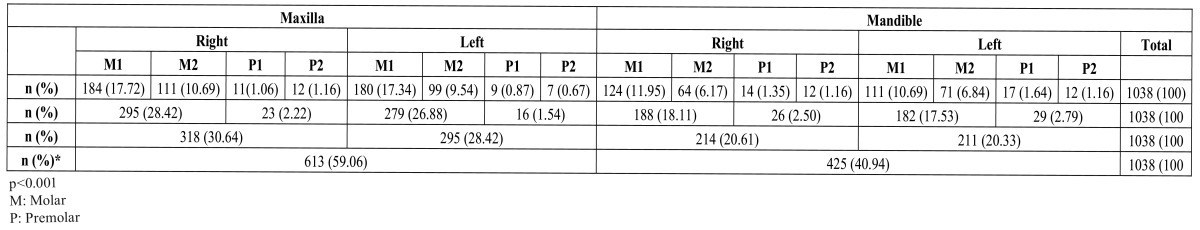
The occurrence of pulp stones in each tooth type, arch, and location.

presented rates based both on the number of patients and teeth. On the basis of the number of patients we found the rate of prevalence to be 57.6%, which is within the reported range in the literature (15,16). Moreover, the rate of prevalence reported in our study is slightly higher than a recently reported study performed in the same region of Turkey (16).

On the basis numbers of teeth examined in previous studies, pulp stones were detected in 1,058 (14.8%) out of the 6,228 teeth examined in a teenage group of 515 subjects (7). In another study the prevalence of pulp stones was found to be 22.4 % in 1,028 of 4,573 teeth examined (15). Renjitker et al. found the prevalence to be 10.1 % in 333 out the 3,296 teeth examined (8). Another report related to the prevalence of pulp stones showed pulp stone incidence to be 4.8 % in 747 out of the 15,326 teeth examined (16). In the present study, we found that the prevalence of pulp stones was 15 % in 1,038 of 6,926 teeth examined. 

Pulp stones were more frequently encountered in females than in males with significant differences between the genders in each tooth type and arch (p<0.001). The prevalence of pulp stones noted in females and males in this study agrees with previous studies that it is greater in women (6,7). In the literature, bruxism which causes longstanding irritation on dentition was thought to be the reason of this difference because it is more prevalent in women (16). The statement that the effect of bruxism increases the prevalence of pulp calcifications in women should be investigated in further studies. 

In the literature it was reported that subjects older than age 60 years had significantly higher prevalence of pulp stones in compared to younger age groups (9,11). However age was not found related with pulp stones in our subjects. This may be due to the fact that, the majority of the patients (63.49 %) were in the second and third decades of their lives and we had only a few patients older than fifty years in our study. 

The occurrence of pulp stones was more frequently found in the maxilla than in the mandible in each tooth type and location (right- left) in the present study. These results are not in agreement with previous studies (7,15). However, there are studies indi-cating higher frequency of pulp stones in the maxilla (8). The prevalence of pulp stones in the present study was found to be higher in both genders in the first molars than in the second molars and premolars; this finding also confirms the results of other studies (11). This result may be related to the fact that the molars are the largest teeth in the arch, provide a better supply of blood to the pulp tissue and have the strongest chewing force in the arch. This may lead to greater precipitation for calcification.

The detection of pulp stones can be observed by dental radiograph. However, to detect these calcified structures, the diameter of which can be bigger than

**Table 3:**
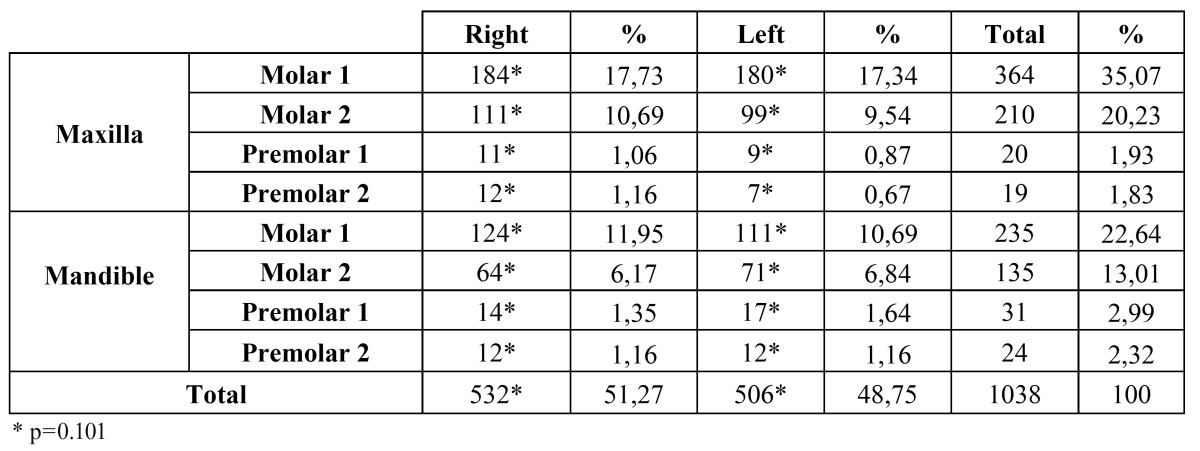
The distribution of pulp stone according to dental arches and location.

**Table 4:**
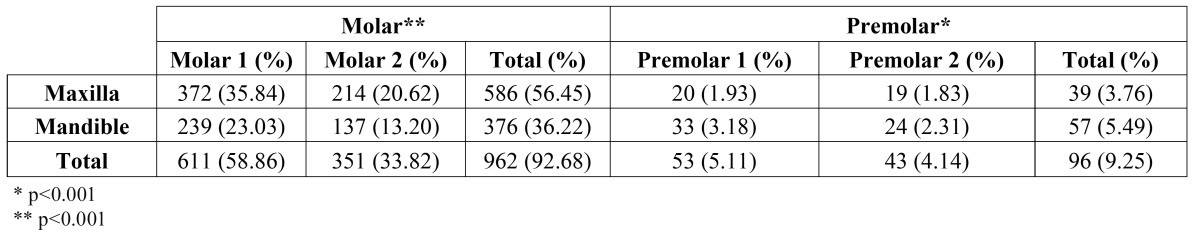
The distribution of the molar and premolar teeth with pulp stone according to dental arches in each tooth type.

200 цm, proper radiographic techniques must be used. The bitewing radiographic technique which was used on in this study is a better radiographic method than the periapical and panoramic techniques, since distortion can occur in the picture in the latter, while in the paralleling technique a more standard picture can be obtained by having the central beam perpendicular to the long axis of the teeth (7). Therefore the bitewing radiographic technique was used and the inter observer agreement value was almost perfect in determining pulp stones in the present study.

The etiological factors for the formation of pulpal calcifications are not well understood. Age, gender, systemic disease, and long-term irritation such as deep caries and restorations have been proposed as possible implicated factors in the development of pulpal calcifications (18,21). The pathological effect of irritation by the microorganisms of dental caries on the pulpal tissue can cause a vascular wall injury, resulting in the deposition of calcium salts within the tissue (7). Although the currently held clinical view is that pulp stones have no clinical significance, they lead to complications when endodontic therapy is needed; this may lead to hindering canal location and negotiation. Authors also reported a correlation between pulpal calcification and cardiovascular disease and those subjects with a history of cardiovascular disease were found to have an increased incidence of pulp stones in asymptomatic vital pulps, compared to subjects with no history of cardiovascular disease (9,18,22). This shows that pulp stones found incidentally in the pulp tissue play an important role in the diagnosis of a serious underlying disease or condition. In addition, in forensic dentistry, the radiographic matching of pulp stone configurations, along with other features recorded in dental records, may provide valuable information in the identification of deceased persons (3). In addition, based on the patient’s dental data including radiographs it is probable that the presence of pulp stones in pulp tissue may be associated with heart attack related death. Therefore, further research related to pulp stones may contribute additional information to the field of forensic medicine.

The incidence of pulp stones was found to be 15% in a Turkish population, which is in agreement with previous studies on the subject. Pulp stones are not only incidental radiographic findings of the pulp tissue but may also be an indicator of some serious underlying disease.
